# First Gradually, Then Suddenly: Understanding the Impact of Image Compression on Object Detection Using Deep Learning

**DOI:** 10.3390/s22031104

**Published:** 2022-02-01

**Authors:** Tomasz Gandor, Jakub Nalepa

**Affiliations:** 1 Polish-Japanese Academy of Information Technology, Koszykowa 86, 02-008 Warsaw, Poland; 2 KP Labs sp. z o.o., Konarskiego 18C, 44-100 Gliwice, Poland; nalepa@kplabs.pl or; 3 Department of Algorithmics and Software, Faculty of Automatic Control, Electronics and Computer Science, Silesian University of Technology, Akademicka 16, 44-100 Gliwice, Poland

**Keywords:** deep learning, object detection, image compression

## Abstract

Video surveillance systems process high volumes of image data. To enable long-term retention of recorded images and because of the data transfer limitations in geographically distributed systems, lossy compression is commonly applied to images prior to processing, but this causes a deterioration in image quality due to the removal of potentially important image details. In this paper, we investigate the impact of image compression on the performance of object detection methods based on convolutional neural networks. We focus on Joint Photographic Expert Group (JPEG) compression and thoroughly analyze a range of the performance metrics. Our experimental study, performed over a widely used object detection benchmark, assessed the robustness of nine popular object-detection deep models against varying compression characteristics. We show that our methodology can allow practitioners to establish an acceptable compression level for specific use cases; hence, it can play a key role in applications that process and store very large image data.

## 1. Introduction

Real-life applications of object detection, such as intelligent video surveillance systems, faced a number of practical challenges. One critical challenge is how to handle a large volume of image data efficiently. Since city surveillance systems are spread geographically and include hundreds (or even thousands) of cameras, processing captured data requires compression, usually in a lossy manner. Such lossy compression ultimately degrades the quality of images because it discards a portion of the contained information. Therefore, adjusting the compression settings leads to a trade-off between image quality and storage/transfer requirements and constraints.

Object detection is a prominent task in computer vision. It has received much research attention because of its cornerstone role in many practical applications ranging from personal photography to security and surveillance. Additionally, 2D-object detection can play a key role in many other areas, such as 3D sensing (e.g., when combined with LIDAR data [1]), or autonomous driving [2], in which detecting tiny objects suchas traffic signs in real time is crucial [3]). Although there are numerous classical machine learning approaches for object detection, deep-learning object detectors are advantageous compared to other kinds of algorithms for the following reasons:

- They offer a high object-detection performance that outperforms classical approaches [4];
- They can be trained to detect new classes of objects without programming new algorithms or feature extractors in a human-dependent manual way [5]; and
- Hardware acceleration to address their substantial computational needs is readily available, thus allowing end-to-end training of large models.

Although there have been attempts to verify the impact of lossy image and video compression on the performance of deep convolutional architectures applied in various computer vision tasks (e.g., human pose estimation, semantic segmentation, object detection, action recognition, or monocular depth estimation [6,7]), the quality of images after lossy compression was considered primarily with human perception in mind [8,9,10]. We follow the former research pathway, and our main objective is to understand how image compression affects the performance of deep-learning models for object detection.

### 1.1. Related Work

Most research into object detection from image data does not concurrently take into account image quality and lossy compression, or their impact on object detection performance. However, there some works on deep convolutional neural networks (CNNs) performance under different conditions, sucha as quality degradation resulting from input data compression. (Note that compressing CNNs and elaborating resource-frugal deep models is another interesting research area, for which we are aiming to obtain compact models that occupy less memory and infer faster, ideally without degrading the abilities of the algorithm [11,12]).

Dodge and Karam [13] investigated the influence of image quality on the performance of image classification, which is similar to object detection but without the localization requirement. One of the methods of quality degradation was lossy image compression, using JPEG and JPEG2000. The study was based on the ImageNet 2012 1000-class dataset, specifically on 10,000 images drawn from the ILSVRC 2012 validation set [14]. Four deep architectures, two variants of AlexNet alongside the larger VGG-16 network and GoogLeNet [15], were exploited in the experimental study that covered five types of image distortions: (i) additive Gaussian noise, (ii) blur via convolution using a Gaussian kernel, (iii) contrast reduction via blending with an uniform gray image with a varying blending factor, (iv) JPEG compression at different quality levels (reflected by the Q parameter), and (v) JPEG2000 compression with a different target peak signal to noise ratio (PSNR). The authors measured Top-5 classification accuracy (the percentage of classifications where the correct class was among the five most confident predictions), as well as the strict (Top-1) accuracy. The experiments indicated a significant influence of blurring, together with a medium influence of noise and a high robustness against the contrast degradation. Both image compression methods were found to have an impact on the classification performance. For all considered models, the accuracy did not decrease significantly for quality levels from 20 to 100.

In [16], the authors described methods for generating additive, seemingly random, noise with low amplitude, causing models to classify modified images wrongly. Although such alterations are perceivable by humans, they do not affect object recognition capabilities. This showed that the image quality can be regarded differently for human perception and for (at least some) deep CNN models. This observation was explored further in [13] for the image classification task. In the work reported here, we tackle the problem of understanding the influence of lossy compression on deep-learning object detection, which remains an open question in the literature.

### 1.2. Contribution

We investigate the influence of the JPEG compression on the performance of CNNs for object detection. This compression method is widely used in various real-life applications, such as digital photography or document archiving. Moreover, other lossy compression algorithms, including video encoders, are based on the same principles [17]. The insights learned from our study can be generalized over many other compression techniques as well. Our contribution centers around the following points:

- We devise a fully reproducible computational study to thoroughly assess the influence of varying compression levels on the performance of a representative collection of models for object detection (including one-stage and two-stage detection pipelines), using a well-known validation dataset [18].
- We analyze the changes in performance using both an aggregated performance score, as well as separate metrics of recall and precision to allows us to observe differences in behaviors between models in detail.
- We show how different architectures behave with respect to the confidence threshold, and we present the examples of high and low sensitivity to such threshold, which needs to be taken into account while balancing precision and recall.
- We examine the influence of object size (quantified as the object’s area in pixels) on the robustness of object detection.

Our findings have a practical application in systems using deep CNNs for object detection, which uses lossy compression for image transfer or storage. The insights concerning the nature of the trade-off between the compression level and detection performance can lead to better decisions about the underlying compression parameters and to a reduction in data storage requirements, while maintaining an acceptable performance of the deep learning-powered object detectors.

### 1.3. Paper Structure

The remainder of this paper is organized as follows. Section 2 describes the materials and methods used in our study. Here, we discuss the JPEG compression algorithm, together with the deep-learning-powered object detection and the metrics that are commonly used to quantify the performance of such techniques. We also elaborate on the benchmark dataset and the models exploited in our study. Section 3 presents and discusses the experimental results, and Section 4 concludes the paper.

## 2. Materials and Methods

### 2.1. JPEG Image Compression

**Figure 1:**
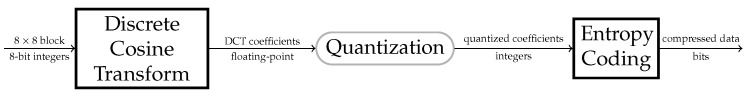
The JPEG compression pipeline. The lossy step is indicated as a rounded block.

A high-level JPEG image data compression flowchart is rendered in Figure 1. We discuss its pivotal steps in more detail in the following subsections.

#### 2.1.1. Block Transform

The lossy compression used in the JPEG standard is based on the discrete cosine transform (DCT) [19]. The forward DCT (FDCT), also known as DCT-II, processes an 8×8 block of samples (f(x,y)∈[0,255]) producing an 8×8 block of DCT coefficients F(u,v) using the following formula [20]:

(1) $$F\left(u,v\right)=\frac{1}{4}C\left(u\right)C\left(v\right)\left[\sum_{x=0}^{7}\sum_{y=0}^{7}f\left(x,y\right)\cos\frac{(2x+1)u\pi }{16}\cos\frac{(2y+1)v\pi }{16}\right],$$

where C(u) and C(v) are the normalization constants defined as follows:

(2) $$C\left(u\right),C\left(v\right)=\left\{\begin{array}{cc} 1/\sqrt{2}, & \mathrm{if}\;u,v=0,\\ 1, & \mathrm{otherwise}. \end{array}\right.$$

This transform can be reversed using the associated inverse DCT (IDCT), also known as DCT-III, which is defined as follows:

(3) $$f\left(x,y\right)=\frac{1}{4}\left[\sum_{u=0}^{7}\sum_{v=0}^{7}C\left(u\right)C\left(v\right)F\left(u,v\right)\cos\frac{(2x+1)u\pi }{16}\cos\frac{(2y+1)v\pi }{16}\right].$$

#### 2.1.2. Quantization

The output of DCT is quantized—each DCT coefficient in the 8×8 block is divided by its corresponding quantization table (QT) entry Q(u,v)∈[1,255] rounded to the nearest integer. This operation is only approximately reversed during the decompression process: before the inverse transform, the coefficients are multiplied by their QT entries. The larger the divisor in the QT becomes, the lower the number of the discrete quantized coefficient values that can be generated is. Finally, the DCT coefficients that are smaller than a half of the QT entry become zero.

#### 2.1.3. Setting the JPEG Quality

**Figure 2:**
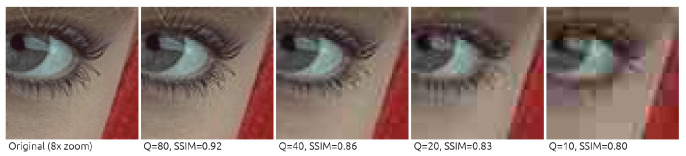
The JPEG compression quality. An example 64×64 image section compressed with the selected Q values, together with the corresponding SSIM [21]. For visibility, the image section was zoomed 8× using the nearest-neighbor interpolation. Source: http://r0k.us/graphics/kodak/kodak/kodim04.png, license: CC0, accessed on 19 December 2021.

The Q parameter, which is used to control image quality in JPEG compression, is an integer in the range [1,100], with the smaller values indicating lower quality and smaller output size, and higher values corresponding to better quality at the cost of size (hence, “weaker” compression)—see Figure 2. Interestingly, the value of 100 does not correspond to the lossless compression, but to the configuration that introduces the smallest possible information loss, which is achieved when there are only ones in the QT. For Q=1, all divisors in the QTs are equal to 255. The QTs used in this research are available at https://github.com/tgandor/urban_oculus/tree/master/jpeg/quantization (accessed on 19 December 2021).

The JPEG data compression ratio may vary since it depends on image content, e.g., a single-level 8×8 block needs to store one DCT coefficient, but high-frequency areas may have 64 non-zero coefficients even after quantization. Table 1 shows the compression ratio statistics for a set of the Q values computed on the COCO val2017 set.

#### 2.1.4. Relation between the Q Parameter and the Image Quality

Image quality can be defined in multiple ways, one of which uses full-reference image quality metrics (FIQMs). They can be applied if the reference image is available, so the similarity can be expressed numerically. Such metrics include the mean squared error (MSE) and the root mean square error (RMSE), which depends on the dynamic range of the image. Still, they are useful in various scenarios, e.g., they can be exploited as loss functions while training image autoencoders. The peak noise-to-signal ratio (PSNR) is measured in decibels and is computed as

(4) $$\mathrm{PSNR}=10\cdot \log\left(\frac{{\mathrm{MAX}}_{I}^{2}}{\mathrm{MSE}}\right).$$

It is worth mentioning that PSNR is used in the JPEG2000 algorithm to control the compression quality, but it suffers from several limitations that were pointed out by Wang and Bovik [22]. A popular FIQM which overcomes these shortcomings is the structural similarity index metric (SSIM) [21]. In contrast to MSE, which is computed pixel-wise, SSIM uses a block-wise computation, which is averaged for the entire image. For each block, the metric compares the average pixel value, the standard deviation, and also the co-variance of two blocks, and normalizes the result to [0.0,1.0]. Table 2 gathers the SSIM statistics obtained for the selected Q values over the COCO val2017 set.

### 2.2. Deep Learning in Object Detection

Deep learning has been blooming in the field of object detection [23], and a plethora of techniques benefiting from automated representation learning have been proposed for this task so far [24]. The following subsections discuss such approaches in more detail.

#### 2.2.1. The Object Detection Pipeline

**Figure 3:**
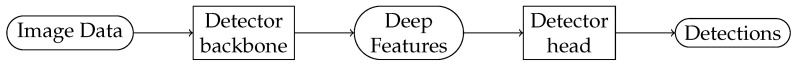
A high-level object detection pipeline that exploits deep learning. The deep CNN-based object detectors used in this study consist of a backbone and a head. The rectangles represent the functional elements, whereas the rounded rectangles are their input and output data.

A high-level flowchart of the object detection pipeline that exploits deep learning is rendered in Figure 3. Such deep learning-powered models include

- One- and two-stage detectors (also referred to as the dense and sparse detectors), and
- Detectors, those that use a feature pyramid built on top of the backbone and those that exploit the final convolutional layer of the backbone.

#### 2.2.2. The Backbone for Feature Extraction

**Figure 4:**
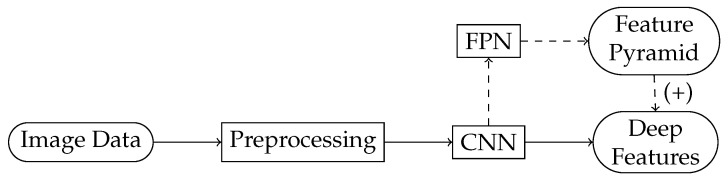
The backbone of an object detector. This part of the network extracts the deep features.

The object detection pipeline commonly starts with feature extraction, which may be followed by feature selection [25]. A flowchart of the backbone that extracts features, together with an optional feature enhancement pathway, is shown in Figure 4. The enhancement is achieved by building a feature pyramid network (FPN) [26], which is a fusion of high- and low-level features [27].

Before the features are actually extracted, the input images are pre-processed, a step that often includes their resizing so they can be fed into the input layer, and standardization. The backbones are usually taken directly from a well-established deep image classifier [28,29]. The most popular backbones encompass the ResNet [30], ResNeXt [31], DarkNet [24], MobileNet [32], and EfficientNet [33] architectures.

#### 2.2.3. Single-Stage Detectors

Single-stage detector architectures (also referred to as the single-shot [24,34] and dense detectors [35]) perform prediction directly on the output features of the backbone network. Single-shot detectors process the input image (i.e., extract the features) only once [24], which was not the case in the earlier two-stage detectors. Additionally, *every* point of the final feature map (or feature maps for feature pyramids) can potentially detect a specified number of objects. Finally, localization and classification tasks can be handled by two separate sub-networks, as in RetinaNet [35], or a single fully convolutional network, as presented in [36].

#### 2.2.4. Two-Stage Detectors

**Figure 5:**
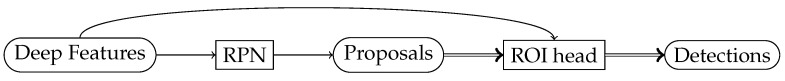
A two-stage detector head, also referred to as the sparse detectors.

The two-stage detectors (also referred to as the sparse detectors) are built with two functional blocks (as shown in Figure 5):

- The region of interest (ROI) proposal mechanism, which generates the locations (boxes) in the image (where an any-class object can be found); when implemented as a neural network, it is known as the region proposal network (RPN).
- The ROI heads, which evaluate the proposals, producing detections.

In the two-stage detection approach, the detection occurs only in a limited number of regions, which were produced by the RPN, and not across the entire image. Therefore, the most important quality metric related to the RPN is its recall.

A ROI head performs the second stage of the sparse-object detection. It takes a proposal from RPN together with the deep features from the backbone. The features relevant for a given region are processed through an operation called the ROI pooling and are fed to the networks that localize and classify the objects.

### 2.3. Performance Metrics for Object Detection

This section describes the performance metrics that apply to the task of object detection. Here, we show which count as true positive (TP) and false positive (FP) objects, how the results are aggregated over a benchmark dataset, and which parameters (thresholds) can be specified for the metrics.

#### 2.3.1. Assessing a Single Detection

A single detection returned by the model needs to be categorized as a TP or FP. To determine this, the intersection over union (IoU) is commonly used. This value is the result of dividing the areas of the ground-truth and predicted box intersection (or zero if the boxes are disjoint) by the area of their union.

The threshold value for IoU, denoted as $T_{IoU}$, is a parameter of the object detector evaluation: a detection is treated as a TP, if there exists a ground truth (GT) box for the same class with an $\mathrm{IoU}\geq T_{IoU}$; otherwise, it is treated as a FP. The choice of the $T_{IoU}$ value strongly influences the quantitative results. Thus, only the values obtained with the same $T_{IoU}$ should ever be compared. Commonly, the metrics found in the literature specify the threshold used—too low can lead to an over-optimistic evaluation and an incorrect assignment to the GT boxes, and too large a threshold may cause many correct detections to be rejected, especially if the GT boxes are not accurate. The lowest widely used $T_{IoU}$ is 0.5, followed by 0.75 when the localization accuracy requirements are strict. Each detection has a confidence *p*—we apply the confidence threshold $T_{c}$ and process the detections with $p\geq T_{c}$.

#### 2.3.2. Detecting the Unlabeled Objects: Crowds

The object detection datasets may include some annotations designated as “crowd”. These regions include many objects of the same class without individual object annotations. We record the number of such detections, and refer to them as the “extra” detections (EX).

#### 2.3.3. Precision, Recall and the F1-Score

Once the number of TP and FP detections was determined for the specific values of $T_{IoU}$ and $T_{c}$, we calculated the precision (positive predictive value, PPV), which is the ratio of TP and the total number of detections:

(5) $$\mathrm{PPV}=\frac{\mathrm{TP}}{\mathrm{TP}+\mathrm{FP}}.$$

To calculate the recall metric (also called sensitivity, true positive rate, TPR), we additionally exploited the number of objects in GT that were not detected such an a false negative (FN). This metric became

(6) $$\mathrm{TPR}=\frac{\mathrm{TP}}{\mathrm{TP}+\mathrm{FN}}.$$

The F1-score aggregates TPR and PPV into a single value in the range [0,1], by using the harmonic mean:

(7) $$F1={\left(\frac{{\mathrm{TPR}}^{-1}+{\mathrm{PPV}}^{-1}}{2}\right)}^{-1}=2\cdot \frac{\mathrm{PPV}\cdot \mathrm{TPR}}{\mathrm{PPV}+\mathrm{TPR}}=\frac{2\cdot \mathrm{TP}}{2\cdot \mathrm{TP}+\mathrm{FP}+\mathrm{FN}}.$$

The $T_{c}$ parameter can be used to tune the above performance metrics—increasing the threshold potentially increases precision at the cost of recall, and lowering it has the opposite effect.

#### 2.3.4. The Precision–Recall Curve and Average Precision

**Figure 6:**
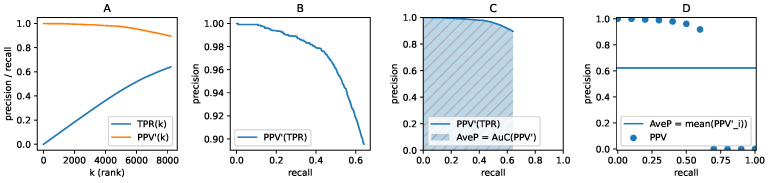
The AP score derived from a precision-recall curve. (**A**) Example plot of recall and monotonic precision on the *k* top confidence detections. (**B**) Monotonic precision plotted against recall. (**C**) AP as the area under the precision-recall curve in the unit square. (**D**) AP as the average of precision sampled at 11 recall values [0.0,0.1,…,1.0]. If a recall value is never reached, the precision becomes zero for that value.

All detections for all images in a benchmark dataset are first sorted by their confidence in descending order. When considering the top *k* elements, the $TP_{k}$ and $FP_{k}$ values can be used to compute the running precision $PPV_{k}=TP_{k}/\left(TP_{k}+FP_{k}\right)$, and recall $TPR_{k}=TP_{k}/GT$, where GT is the number of ground-truth objects in the dataset. $TPR_{k}$ is a non-decreasing series, but $PPV_{k}$ is not monotonic. To convert $PPV_{k}$ into a non-increasing curve, we used $PPV_{k}^{\prime }=\max_{i\geq k}PPV_{i}$. To efficiently compute AP, we sampled the precision by recall (Figure 6D), and therefore we obtained

(8) $$PPV^{\prime }\left(r\right)=\left\{\begin{array}{c} PPV_{k},k=\min\left\{k\;|\;TPR_{k}\geq r\right\}.\\ 0,r>\max TPR_{k} \end{array}\right.$$

The AveP in this approximation becomes the arithmetic mean:

(9) $$AveP=\frac{1}{\left|R\right|}\sum_{r\in R}PPV^{\prime }\left(r\right),R=\left[0,0.1,\ldots ,1.0\right].$$

Finally, the set of the recall samples R is evenly spaced from 0 to 1, usually by 0.1 (as proposed in [37]) or by 0.01 (as exploited in [18]). The code for evaluating the AP metric is available at https://github.com/cocodataset/cocoapi/ (accessed on 19 December 2021).

#### 2.3.5. The Performance Metrics Selected for This Study

After each step in the image degradation, the objects are detected using each investigated model, and the performance metrics are computed. The parameters were set as follows: $T_{c}=0.5$ (the confidence *p* cutoff), and $T_{IoU}=0.5$ (for the metrics using a single IoU threshold except mAP_.75_, for which $T_{IoU}=0.75$), and $T_{IoU}\in \left[0.5,0.55,\ldots ,0.9,0.95\right]$ for AP. The following object detection performance metrics were evaluated for all experiments:

- TP: the number of true positive detections,
- FP: the number of false positive detections,
- EX: the number of the “extra” (crowd) detections,
- PPV: the overall precision of the detections,
- TPR: the overall recall of the detections,
- F1: the overall F1-score,
- AP: the overall AP metric,
- AP_s_, AP_m_, AP_l_: AP separately for small (below $32^{2}=1024$ pixels of area), medium (between 1024 and $96^{2}=9216$ pixels) and large (above 9216 pixels) objects,
- mAP_.5_, mAP_.75_: the mean average precision for two different $T_{IoU}$ values.

### 2.4. Qualitative Assessment of the Detection Performance

The 5000 images included in the val2017 dataset, multiplied by 100 quality settings and 9 models gives 4.5M of possible images with detections, which is infeasible to analyze manually. However, we analyzed a subset of the detections qualitatively, and proposed names for the unwanted behavior of the detectors. The errors encompass

- omission of an object (FN), the most common error,
- wrong classifications (a bounding box around an object with the wrong category returned, sometimes alongside a correct detection of that very object),
- mistaken objects (detecting real objects with a correct bounding box, but of a category not present in the GT),
- detections of unrelated objects at random places in the image (“halucinating”).
- loss of bounding box accuracy,
- selecting only part of an object or having multiple selections of the object (loss of continuity),
- one box covering multiple objects (cluster) or parts of different objects from the same category (chimera).

### 2.5. Reproducibility Information

All Python code and Jupyter notebooks associated with the study were published at https://github.com/tgandor/urban_oculus (accessed on 19 December 2021). The input data is available for download at https://cocodataset.org/ (accessed on 19 December 2021), and the raw output data (detections in the JSON format) was deposited in a public data repository at https://doi.org/10.7910/DVN/UPIKSF (accessed on 19 December 2021).

#### 2.5.1. Benchmark Dataset

There is a plethora of datasets for object detection [23], such as PASCAL VOC, ImageNet, Open Images and MS COCO. We exploited the validation subset of the COCO Detection Challenge 2017, which is called val2017 for short. It consists of 5000 images of objects in natural environments. These are known as non-iconic images [18], in contrast to iconic images which are typically used for image classification. There were 80 object categories and 36,781 annotated objects in total. The number of objects in each category was uneven: the top 3 of them were people (11,004), cars (1932), and chairs (1791), while the least represented two classes contained only 11 and 9 object instances. The original JPEG quality of the images had the following distribution: Q=96:3540, Q=90:1414, Q=80:46. Finally, there were 134 grayscale images.

#### 2.5.2. The Investigated Deep Models

For object detection, we used nine pre-trained deep models taken from the Detectron2 [38] Model ZOO available at https://github.com/facebookresearch/detectron2/blob/master/MODEL_ZOO.md (accessed on 19 December 2021). The models were given the following identifiers: R101, R101_C4, R101_DC5, R101_FPN, R50, R50_C4, R50_DC5, R50_FPN, X101 (Table 3). This choice of models wais comprehensive, and covered both one-stage (RetinaNet) and two-stage (Faster R-CNN) detectors, as well as different variants of Faster R-CNN (with and without the feature pyramid). As the backbones, we used two ResNet depths (50 and 101 layers0, and there was one backbone using ResNeXt-101 (X101). The non-FPN Faster R-CNNs had two kinds of backbones: the first (C4) used a standard ResNet, and the other (DC5) exploited dilated convolutions (DC). Finally, both RetinaNets included a FPN. All the models were trained on the train2017 dataset [18], which was the training subset of the COCO Detection Challenge 2017. It encompassed the same 80 categories as val2017, but there were many more images (117,266) and object annotations (849,949). The stochastic gradient descent optimizer with a 0.9 momentum value, 270,000 iterations, and 16 images per batch (36 epochs in total) was used to train the deep models.

#### 2.5.3. Image Degradation

This step performs the process of compressing all images from the benchmark set to a certain quality setting *Q*. For this task, the mogrify program from the ImageMagick suite (available at https://imagemagick.org/; accessed on 19 December 2021) was used throughout the computational experiments. The following command was run for the degradation:

$ mogrify -verbose -quality <Q> datasets/coco/val2017/*.jpg

Since this operation is deterministic, there was no need to publish the degraded images for reproducibility. The set of the *Q* parameter values was all integers from the range 1,2,⋯,100. Importantly, no spatial transformations were applied to the input images; hence, the object locations and classes remained unchanged throughout the experiment.

## 3. Experimental Results

### 3.1. The Baseline Results

The inference was first executed for all the models on unchanged images (the baseline), with $T_{c}=0.5$. These baseline results are presented in Table 4.

#### 3.1.1. The AP and Related Metrics

For the metrics based on the AveP, AP, mAP, and per-size AP values, X101 was the dominant model except for AP_l_, where the simple R101_C4 model achieved 1.5 percentage points more. However, the second places behind X101 in AP and mAP were specific to the metric:

- For AP, the ResNet-101 Faster R-CNNs were only 0.1% from each other, with the following order of its variants: C4, FPN, DC5.
- For mAP_.5_, the order was DC5, C4 (−0.5%), FPN (−0.8%).
- For mAP_.75_, the order was FPN, DC5 (0.6%), C4 (−0.1%).

This meant that the benefits of FPN were manifesting themselves with a higher precision of the bounding boxes, which improved mAP for higher $T_{IoU}$, and thus also AP. The RetinaNets fell behind the two-stage models in these metrics because of low TPR values (around 50%) compared to the 63–68% range of Faster R-CNNs.

#### 3.1.2. Counting the Objects (TP, FP, EX), Recall and Precision

In the baseline results from Table 4 for the detected object counts, the TPR and PPV of the detection were more nuanced then the AP-related ranking. The largest numbers of objects were found by the classic Faster R-CNNs (about 24.5k or 67–68% TPR). This was closely followed by X101 with 66%, and two other FPN-based models achieving 64–65%. The ranking was concluded by the RetinaNets, which achieved only 50–52% for detecting 18–18.5k objects. Interestingly, the precision ranking was exactly reversed. The RetinaNets returne only about 4.3k FP, which was less than 20% of their total detections (81% PPV). This precision was more than 10% higher than that of the next group (FPN-based models), which had precision values in the range of 67–70%. The non-FPN two-stage models were another 10% below that, with a PPV ranging from 57 to 59%. The EX metric, which counted the additional objects in the “crowd” regions, was similar to the TPR, but there were greater differences between the RetinaNets (about 800 detections), FPN models (2.5–2.8k detections) and non-FPN models 4.5–4.7k detections). Surprisingly, the ResNet-50 Faster R-CNNs produced even more EX detections than those based on ResNet-101.

#### 3.1.3. Discussing the Impact of $T_{c}$

**Figure 7:**
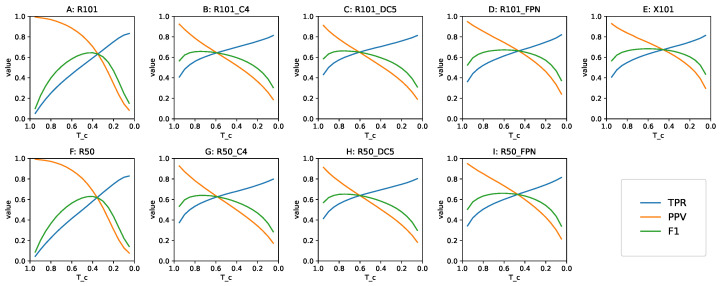
Baseline TPR, PPV and F1 as a function of $T_{c}$. Top row: 101-layer backbone models, bottom row: 50-layer backbone models. (**A**) RetinaNet achieves low values of each metric at respective $T_{c}$ extremes, and a narrow range of the best F1. (**B**,**C**) Faster R-CNN without FPN exhibits similar behavior regardless of using dilated convolutions, the best F1 is returned at a higher $T_{c}$. (**D**,**E**) Faster R-CNN with FPN maintains the balance between TPR and PPV in a wide $T_{c}$ spectrum, with the ResNeXt backbone having better metrics and a symmetrical F1 curve. (**F**–**I**) Models with backbones that are less deep are analogous to their larger equivalents.

For practical applications such as in video surveillance, when detected objects cause a resource-consuming intervention, an appropriate $T_{c}$ value needs to be determined in advance. When the benefit of GT annotations is not present, the risk of missing objects needs to be balanced against the cost of human attention dedicated to reviewing FP, by means of setting a right $T_{c}$ value. The $T_{c}=0.5$ is a good simulation of such a situation, because it expresses the greater prior probability of a detection being correct than false. Having collected all detections with the confidence p≥0.05, we examined the baseline models’ behavior in a wide range of the $T_{c}$ values. Figure 7 shows the precision, recall and F1-score of each model in our study as a function of $T_{c}$.

The $T_{c}$ value with the best F1-score is not necessarily the best threshold for any given detection task, but it informs us about the trade-off between the TPR and PPV. When the shape of F1 as a function of $T_{c}$ is steep, with a small region of values close to maximum, it means that the corresponding model is highly sensitive to the choice of threshold. A flatter shape, with a plateau in the neighborhood of the maximum, indicates that the choice of $T_{c}$ may be more arbitrary, and favoring either the TPR or PPV does not disproportionately affect the other metric. Looking at Figure 7, we can confirm that $T_{c}=0.5$ was an acceptable choice for all the models in this study.

### 3.2. Detection Results on Degraded Images

**Figure 8:**
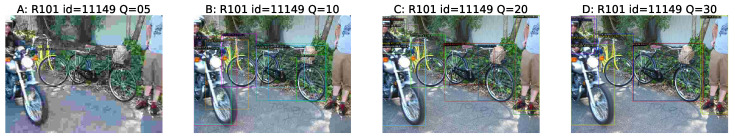
The R101 detection examples: bicycles and motorcycles. (**A**) Q = 5: the single FP is a box around parts of two different *bicycle* objects (chimera). (**B**) Q = 10: no *person* detected, multiple *bicycle* FPs, one *bicycle* detected correctly, the other with extra detections of its parts. The *motorcycle* detected both correctly, and falsely as *bicycle*. (**C**) Q = 20: both *person* objects detected, with repeated detections of one, similar situation for *bicycle*. (**D**) Q = 30: *person* and *bicycle* correctly detected, the *bicycle* FP includes part of the first bicycle and all of the visible *motorcycle*. Source: http://images.cocodataset.org/val2017/000000011149.jpg, license: CC-BY, accessed on 19 December 2021.

**Figure 9:**
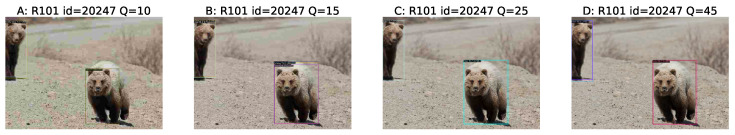
The R101 detection examples: two bears. The detector localizes the objects correctly, but there are classification mistakes. (**A**) Q = 10: classified as *teddy bear* and *person*. (**B**) Q = 20: classified as *teddy bear* and *dog* (left) and as *elephant*, *sheep* and *teddy bear* (right). (**C**) Q = 25: one *bear* is correct, the other classified as *dog*. (**D**) Q = 30: finally both *bear* objects are correct. Source: http://images.cocodataset.org/val2017/000000020247.jpg, license: CC-BY, accessed on 19 December 2021.

**Figure 10:**
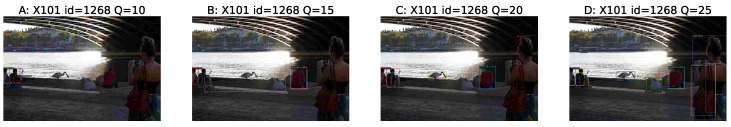
The X101 detection examples: under a bridge. (**A**) Q = 10: two *person* objects; errors: a mistaken *baseball bat*. (**B**) Q = 15: three *person* objects; errors: a hallucinated *train*. (**C**) Q = 20: three *person* objects, one *bird*, one *backpack*, the fourth *person* is annotated with the inaccurate bounding box. (**D**) Q = 25: all *person* objects are correct, the *bird*, a *handbag*, a *boat* are annotated with the inaccurate bounding boxes; errors: one *backpack* is wrongly classified as *suitcase*, there is one mistaken *suitcase*. Source: http://images.cocodataset.org/val2017/000000001268.jpg, license: CC-BY, accessed on 19 December 2021.

**Figure 11:**
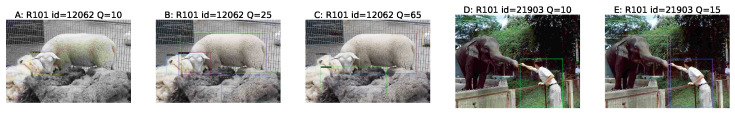
Determining the minimal Q for the correct detection. Two examples of the minimal quality level for detecting objects, or for avoiding a FP. (**A**) Q = 10: a sheep’s head is misclassified as *bird*. (**B**) Q = 25: both GT *sheep* objects are detected (minimal Q), but the head is still mistaken for *bird*, and there is a hallucinated *bird*. (**C**) Q = 65: the central *sheep* is correctly detected, but there is a chimera detection of the head and a pile of wool, there is also a *sportsball* wrongly classified as *cow* (this ball is detected, but never classified correctly, up to Q = 100; it can also be classified as *sheep*). (**D**) Q = 10: both *person* objects are detected, but the *elephant* is omitted. (**E**) Q = 15: the minimal Q for detecting the *elephant*. Source: http://images.cocodataset.org/val2017/000000012062.jpg and http://images.cocodataset.org/val2017/000000021903.jpg, license: CC-BY, accessed on 19 December 2021.

Examples of highly degraded images together with their detections, and—for TP—the GT boxes, are presented in Figure 8, Figure 9 and Figure 10. For every GT object, it is possible to indicate the minimal compression quality at which it was detected by a selected (or by every) model. Surprisingly, there are objects that were detected by all investigated deep-learning detectors even at Q = 1. Conversely, there were cases where the detector made systematic errors (wrong classification or hallucinating the object), up to a certain quality, above which we could observe the correct behavior. Two examples of images demonstrating the “minimal Q” are gathered in Figure 11.

### 3.3. Performance Metrics as a Function of Q

Considering the performance metrics as the functions of Q allowed us to analyze the rate of change by taking a discrete derivative and plot the metrics against the compression quality. In the following subsections, we discuss this in more detail.

#### 3.3.1. The Precision, Recall and F1 Metrics

**Figure 12:**
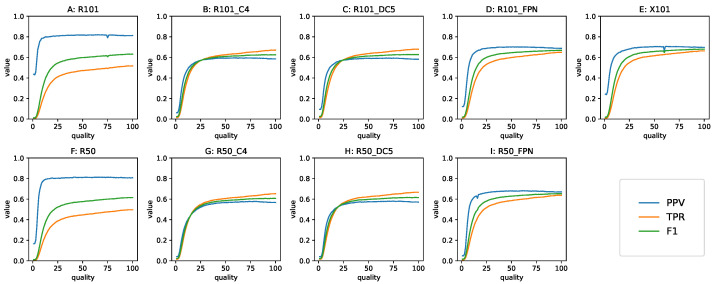
TPR, PPV and F1 as a function of Q at $T_{c}=0.5$. Top row: the 101-layer backbone models, bottom row: the 50-layer backbone models. (**A**,**F**) RetinaNet exhibits high and constant PPV, (**B**,**C**,**G**,**H**) Faster R-CNN without FPN exhibits similar behavior regardless of using dilated convolutions, TPR and PPV are close in a wide range of Q, and recall is better than precision, (**D**,**I**,**E**) Faster R-CNN with FPN exhibits high PPV even for Q<25 while TPR is above RetinaNets and below non-FPN Faster R-CNNs.

The metrics dependent on $T_{c}$ are presented in Figure 12. Here, we can appreciate the near constant value of precision across the range of Q. As a consequence of that, the general decline in performance, in this case measured with the F1 metric, was due to the worsening of recall. The shape of the curves for these metrics depended on the model family. The non-FPN Faster R-CNNs had TPR and PPV values closest to one another, with the TPR starting out higher than the PPV and becoming equal to it near Q = 20. The FPN Faster R-CNN models had higher precision, and recall starts near the precision value, that declined slowly until the turning point. Finally, the RetinaNets had lower TPR values that declined at a comparatively high rate, but they had the highest precision, which was consequently maintained down to low quality values.

#### 3.3.2. The AP, mAP_.5_ and mAP_.75_ Metrics

**Figure 13:**
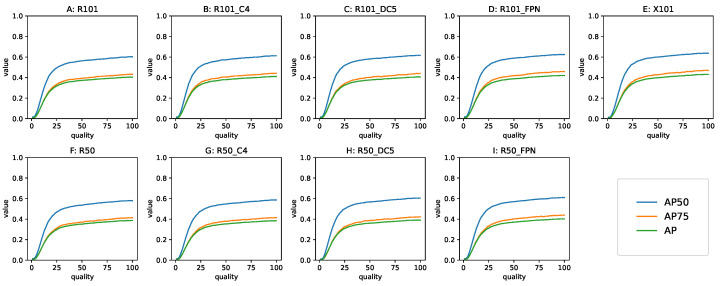
AP, mAP_.5_ and mAP_.75_ as a function of Q. AP50—mAP_.5_, AP75—mAP_.75_. Top row: the 101-layer backbone models, bottom row: the 50-layer backbone models. The results for all the models for AP and related metrics are approximately identical. Note how close mAP_.75_ is to AP. The models: (**A**,**F**) RetinaNet, (**B**,**C**,**G**,**H**) Non-FPN Faster R-CNN, (**D**,**I**,**E**) FPN Faster R-CNN.

The three metrics related to the area under the precision–recall curve behaved as shown in Figure 13. These metrics are averaged, and therefore the curves look smooth. The general shape of AP was the same for all models, manifesting the *first gradually, then suddenly* (a famous E. Hemingway’s quote about the process of bankruptcy) shape. The similarity was visible not only between models, but also between the curves themselves within each detector.

#### 3.3.3. The AP Behavior for Different Sizes of Objects

**Figure 14:**
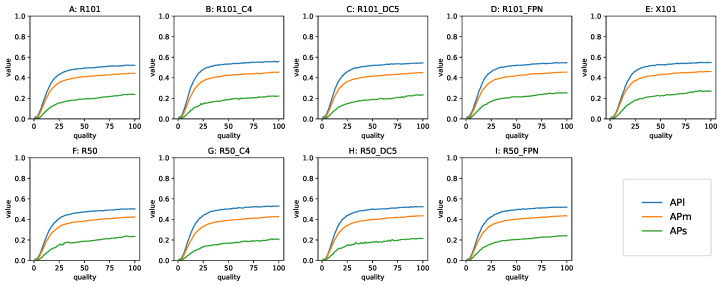
AP for large, medium and small objects as a function of Q. Top row: the 101-layer backbone models, bottom row: the 50-layer backbone models. The results for all the models for the AP metric for different object sizes are almost identical. The models: (**A**,**F**) RetinaNet, (**B**,**C**,**G**,**H**) Non-FPN Faster R-CNN, (**D**,**I**,**E**) FPN Faster R-CNN.

The AP metric computed for small medium and large objects is shown in Figure 14. There was a high similarity across all models, but in contrast to the AP at different $T_{IoU}$, the AP at different sizes had noticeable differences in the curve shape. Specifically, the large objects were robustly detected down to low compression quality, and the shape of the middle-sized objects’ AP curve was similar but with a lower value. This can be related to the influence of the bounding box precision: big objects had high IoU with detections misaligned by a few pixels, and the GT annotations were also not perfect. In the case of small objects, the difficulty of detecting them was apparent in the plots. Not only do they start with AP_s_ approximately half of the AP_m_ of the medium objects, but they declined at a greater rate. This was likely an effect of the lossy compression, which suppresses high frequency signal in the image, which is highly important for analyzing the fine detail of small objects. The AP_s_ curve was visibly noisier than the other curves: the quantization that produces a consistent average effect of the size reduction and quality degradation manifested more randomness in the influence on the small regions, which spanned only few 8×8 compressed blocks.

#### 3.3.4. Analyzing the Derivative of AP with Respect to Q

**Figure 15:**
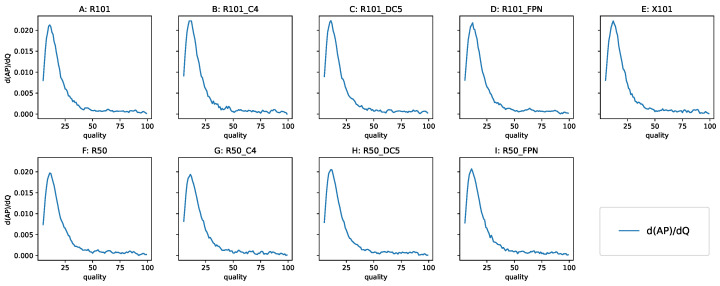
The derivative of the AP with respect to Q, smoothed with an averaging windows of size 5. Top row: the 101-layer backbone models, bottom row: the 50-layer backbone models. This derivative is very similar for all models: the range 40–100 is noisy with a constant running average, below 40, there is an increase in the rate of the AP degradation, with the maximum near Q = 15. The models: (**A**,**F**) RetinaNet, (**B**,**C**,**G**,**H**) Non-FPN Faster R-CNN, (**D**,**I**,**E**) FPN Faster R-CNN.

To confirm the linear behavior of the performance degradation, we analyzed the derivative of the AP with respect to Q. The derivative produced a noisy curve, so we applied smoothing using a running average of five adjacent values—the derivatives are shown in Figure 15. There was a plateau from Q = 100 to Q = 40 (“first gradually”), and then the degradation accelerated (“and then suddenly”). Using the derivative allowed us to pinpoint the Q value where the decline in detection performance started.

#### 3.3.5. The Metric Values for Stronger Compression

Table 5 shows the same set of metrics as the baseline Table 4, but calculated for the dataset degraded by the compression with Q = 25. This value is already below the “turning point”, but the images with this quality are usually good enough for processing by humans, despite visible artifacts. The results showed the precision staying close to the baseline value, and the reduced number of TPs. The number of extra objects was also reduced, as the rate of detecting these objects was comparable to the TPR. Here, we observed that the X101 model was still the best in the AP, mAP_.75_ and AP_s_ metrics (related to the precise localization, especially of smaller objects), but the absolute value of these metrics was low, and the difference relative to non-FPN Faster R-CNNs was smaller. This suggested that the benefits of using the feature pyramid were adversely affected by using too aggressive a compression.

## 4. Conclusions and Future Work

In this paper, we reported our study on the effect of JPEG compression on the performance of deep-learning object detectors based on CNNs. We exploited the COCO val2017 benchmark dataset and collected a wide range of performance metrics for different levels of compression controlled by the parameter Q ranging from 1 (strongest compression, lowest quality) to 100 (weakest compression, nearly lossless). We also established a baseline of metric values for the original dataset. The baseline results were used to characterize the models under testing, including their sensitivity to the confidence threshold value and their trade-off between precision and recall. We performed the qualitative assessment of the detection behavior and introduced a taxonomy of wrong detections: incorrect bounding boxes; wrongly classified, mistaken and hallucinated objects; clusters; and chimera detections.

The experiments showed that the one-stage detectors had a narrower range of admissible thresholds than the two-stage detectors, which were influenced by the threshold but offered a more beneficial trade-off for thresholds further from 0.5. For the metrics obtained over the degraded dataset, we treated them as functions of the parameter Q. For precision and recall at $T_{c}=0.5$, we observed radically different behavior where the precision remained constant regardless of compression quality, as well as a decline in recall that had knee-shaped characteristics and a rapid decrease below Q = 30. The results were consistent for a wide range of $T_{c}$ values with possible shifts in the precision and recall curves, but with the same general shape. We studied the AP metric with its specific cases, the more specialized mAP metrics, and the AP of the large, medium and small objects. The AP, mAP_.5_ and mAP_.75_ curves were similar in all models (with possible differences in scaling, but not general shape), and the mAP_.75_ curve was a good approximation of the more computationally extensive AP. The per-size AP metrics were consistent across the investigated models, with the AP_s_ values having a steeper decline. Therefore, the small objects were more affected by compression. We speculated that this was related to high-frequency information and fine detail, which are not preserved by lossy compression. Finally, we verified the first derivative of the AP curves to find that they are linearly decreased from Q = 96 to Q = 40 (the AP value was approximately constant in the 100–96 range of Q values, but these compression levels were impractical because of an increase in data size). This defined the range of practical compression levels, and the exact Q value for a specific use depended on the recall value that needed to be achieved and the nature of the objects. From a practical perspective, the experimental results helped us draw several important conclusions. Performance decreases with stronger compression following a knee-shaped curve. This curve, as a function of the Q parameter of the JPEG compression, is continuous, so it can be sampled sparsely to save processing time. We observed that some techniques such as FPN lose their benefits over simpler approaches below a certain compression quality. To summarize, the JPEG compression is generally friendly to the deep-learning-powered object detectors, but unlike previous findings about the influence on image classification there was a measurable influence throughout the whole range of the quality settings. This effect ccame from reduced recall while the precision value remained unchanged.

Our study provides a framework for evaluating the influence of image compression on the performance of object detection methods, which can be applied to asses emerging methods for this task. It also opens the door to further research, which encompasses three main directions: broadening the scope of this research, finding the ways to mitigate the effects of image compression on the deep-learning-powered object detectors, and improving compression methods to make them more “friendly” to object detection. The following bullet points summarize a set of potential approaches toward broadening the analysis of the effects of image compression:

- Inclusion of more deep-learning-powered object detection models;
- Expansion of the set of detection performance metrics (e.g., LRP [41], PDQ [42]);
- Incorporation of image quality metrics (based on feature similarity, such as FSIM [43], or salience-aware artifact detection [44], among many others);
- Consideration of related computer vision tasks such as instance segmentation;
- Investigation of other compression algorithms, both transformative (e.g., JPEG2000 [45]) and generative/predictive (e.g., WebP [46], PDE-based methods [47]).

Additionally, the methods that could help overcome compression influence encompass (i) pre-detection quality improvements similar to the super-resolution reconstruction [48,49], (ii) inclusion of compression-degraded images in the training dataset––models could be trained separately over the data with and without quality degradation, or a single model could be built based on the dataset of original and degraded images with the latter treated as the augmented samples, and (iii) building dedicated models for specific ranges of compression quality to be used as ensemble or dedicated models for small and non-small objects, since our results showed that large and medium objects were similar and more robust to the compression effects. These issues constitute our current research efforts that should ultimately lead to more robust deep-learning object detectors ready to be deployed in the wild.

## Figures and Tables

**Table 1:** Descriptive statistics of the compression ratio (as a function of Q) obtained for the COCO val2017 images.

Q	Mean	Std	Min	25%	50%	75%	Max
5	100.51	36.36	1.45	75.61	97.69	125.08	229.79
15	51.34	21.22	1.44	37.01	47.77	61.89	210.04
25	36.45	16.08	1.42	25.97	33.52	43.63	194.23
35	28.96	13.29	1.41	20.43	26.51	34.51	174.91
45	24.31	11.38	1.40	17.13	22.15	28.77	154.25
55	21.46	10.05	1.39	15.26	19.60	25.37	144.08
65	17.64	8.29	1.38	12.54	16.13	20.67	121.30
75	14.78	6.76	1.36	10.61	13.57	17.38	103.31
85	9.60	4.04	1.32	7.05	8.90	11.18	53.47
95	5.56	2.20	1.19	4.15	5.13	6.52	32.92

**Table 2:** Descriptive statistics of SSIM (as a function of Q) obtained for the COCO val2017 images.

Q	Mean	Std	Min	25%	50%	75%	Max
5	0.6271	0.0898	0.1533	0.5741	0.6309	0.6860	0.9657
15	0.7738	0.0637	0.3593	0.7360	0.7802	0.8176	0.9694
25	0.8262	0.0546	0.3900	0.7945	0.8320	0.8643	0.9795
35	0.8549	0.0484	0.4191	0.8276	0.8600	0.8883	0.9760
45	0.8740	0.0436	0.4440	0.8498	0.8785	0.9039	0.9910
55	0.8868	0.0413	0.4674	0.8645	0.8915	0.9150	0.9931
65	0.9047	0.0358	0.5045	0.8865	0.9090	0.9287	0.9881
75	0.9211	0.0331	0.5768	0.9061	0.9256	0.9424	0.9972
85	0.9562	0.0249	0.7819	0.9432	0.9597	0.9757	0.9985
95	0.9947	0.0026	0.9808	0.9931	0.9947	0.9969	0.9996

**Table 3:** The deep object detection models investigated in this study.

Symbol	Description
R101	RetinaNet [35] with ResNet-101 [30] + FPN [26]
R101_C4	Faster R-CNN [39] with ResNet-101 [30]
R101_DC5	Faster R-CNN [39] with ResNet-101 [30] + DC [40]
R101_FPN	Faster R-CNN [39] with ResNet-101 [30] + FPN [26]
R50	RetinaNet [35] with ResNet-50 [30] + FPN [26]
R50_C4	Faster R-CNN [39] with ResNet-50 [30]
R50_DC5	Faster R-CNN [39] with ResNet-50 [30] + DC [40]
R50_FPN	Faster R-CNN [39] with ResNet-50 [30] + FPN [26]
X101	Faster R-CNN [39] with ResNeXt-101 [31] + FPN [26]

**Table 4:** The baseline performance of all investigated deep models ($T_{c}=0.5$).

Model	AP	mAP_.5_	mAP_.75_	AP_l_	AP_m_	AP_s_	TPR	PPV	TP	FP	EX
R101	33.6	47.0	37.2	46.3	37.5	15.3	51.7	**81.1**	18,769	4360	820
R101_C4	38.5	56.3	41.9	**53.6**	42.8	19.1	67.1	58.6	24,373	17,246	4463
R101_DC5	38.3	56.8	42.0	52.1	42.8	19.4	**68.0**	58.2	**24,701**	17,752	4504
R101_FPN	38.4	55.5	42.6	51.2	42.1	20.8	64.9	68.7	23,593	10,751	2605
R50	31.6	44.3	35.2	44.3	34.8	14.1	49.7	80.7	18,043	**4320**	821
R50_C4	35.9	53.6	39.3	51.0	39.8	17.6	65.3	56.8	23,733	18,075	4586
R50_DC5	36.8	55.7	40.5	50.5	41.4	18.2	66.7	57.1	24,244	18,190	**4668**
R50_FPN	36.7	54.1	40.7	49.4	40.1	19.1	63.8	67.0	23,170	11,428	2763
X101	**39.6**	**57.0**	**43.9**	52.1	**42.9**	**22.6**	66.3	69.7	24,073	10,472	2534

The best results are in **bold**, and the second-best are underlined; AP_l_, AP_m_, AP_s_—the AP metric for objects classified as large, medium and small; TPR, PPV—recall and precision at *T_IoU_* = 0.5; TP, FP—true positives and false positives at *T_IoU_* = 0.5; EX—“extra” detection of objects in the crowd regions.

**Table 5:** The performance of all investigated models at Q = 25 ($T_{c}=0.5$).

Model	AP	mAP_.5_	mAP_.75_	AP_l_	AP_m_	AP_s_	TPR	PPV	TP	FP	EX
R101	25.2	36.2	27.8	36.7	28.0	8.8	41.0	**80.9**	14,900	3511	513
R101_C4	30.3	45.5	**32.8**	**45.5**	**33.8**	12.0	56.8	57.1	20,634	15,478	3253
R101_DC5	30.1	**46.6**	32.4	43.5	33.7	12.6	**57.7**	57.3	**20,979**	15,626	3363
R101_FPN	29.2	43.7	31.9	42.1	31.9	13.1	53.1	68.8	19,309	8765	1779
R50	23.2	33.4	25.8	35.0	25.8	7.8	38.7	80.3	14,067	**3458**	470
R50_C4	27.4	42.5	29.6	41.1	30.1	10.7	54.6	52.1	19,842	18,225	3373
R50_DC5	28.4	44.7	30.5	41.0	31.5	12.0	56.5	54.7	20,516	16,974	**3390**
R50_FPN	27.1	42.1	29.5	38.5	30.2	12.1	51.6	66.2	18,761	9575	1784
X101	**30.4**	45.6	**32.8**	42.4	33.5	**13.7**	54.8	68.2	19,914	9269	1736

The best results are in **bold**, whereas the second-best are underlined; AP_l_, AP_m_, AP_s_—the AP metric for objects classified as large, medium and small; TPR, PPV—recall and precision at *T_IoU_* = 0.5; TP, FP—true positives and false positives at *T_IoU_* = 0.5; EX—“extra” detection of objects in the crowd regions.

## Data Availability

The input data are available for download at https://cocodataset.org/ (accessed on 30 19 December 2021), and the raw output data (detections in JSON format) were deposited in the Dataverse public data repository https://doi.org/10.7910/DVN/UPIKSF (accessed on 19 December 2021). All Python code and Jupyter notebooks used in the study were published on GitHub: https://github.com/tgandor/urban_oculus (accessed on 19 December 2021).

## Abbreviations

The following abbreviations are used in this manuscript:

CNN	convolutional neural network
DCT	discrete cosine transform
FDCT	forward DCT
FN	false negative
FP	false positive
GT	ground truth
IDCT	inverse DCT
IoU	intersection over union
QT	quantization table
ROI	region of interest
RPN	region proposal network
TP	true positive
